# Is Carotid Body Physiological O_2_ Sensitivity Determined by a Unique Mitochondrial Phenotype?

**DOI:** 10.3389/fphys.2018.00562

**Published:** 2018-05-16

**Authors:** Andrew P. Holmes, Clare J. Ray, Andrew M. Coney, Prem Kumar

**Affiliations:** Institute of Clinical Sciences, College of Medical and Dental Sciences, University of Birmingham, Birmingham, United Kingdom

**Keywords:** carotid body, hypoxia, mitochondria, nitric oxide, arterial chemoreceptor, O_2_ sensor, metabolism, mitochondrial inhibitors

## Abstract

The mammalian carotid body (CB) is the primary arterial chemoreceptor that responds to acute hypoxia, initiating systemic protective reflex responses that act to maintain O_2_ delivery to the brain and vital organs. The CB is unique in that it is stimulated at O_2_ levels above those that begin to impact on the metabolism of most other cell types. Whilst a large proportion of the CB chemotransduction cascade is well defined, the identity of the O_2_ sensor remains highly controversial. This short review evaluates whether the mitochondria can adequately function as acute O_2_ sensors in the CB. We consider the similarities between mitochondrial poisons and hypoxic stimuli in their ability to activate the CB chemotransduction cascade and initiate rapid cardiorespiratory reflexes. We evaluate whether the mitochondria are required for the CB to respond to hypoxia. We also discuss if the CB mitochondria are different to those located in other non-O_2_ sensitive cells, and what might cause them to have an unusually low O_2_ binding affinity. In particular we look at the potential roles of competitive inhibitors of mitochondrial complex IV such as nitric oxide in establishing mitochondrial and CB O_2_-sensitivity. Finally, we discuss novel signaling mechanisms proposed to take place within and downstream of mitochondria that link mitochondrial metabolism with cellular depolarization.

## Introduction-The Carotid Body and Hypoxia

The mammalian carotid body (CB) is a highly specialized sensory organ derived from the neural crest. The sensory units of the CB are the ‘glomus’ or ‘type I’ cells that respond to a variety of stimuli including hypoxia, hypercapnia, acidosis and hormones thereby allowing the CB to function as a polymodal receptor (Kumar and Bin-Jaliah, 2007; Ribeiro et al., 2013; Thompson et al., 2016). Type I cell activation leads to stimulation of adjacent chemoafferent fibers that relay sensory information into the central nervous system. The physiological consequence of CB stimulation is therefore the initiation of series of systemic protective reflexes characterized by increased ventilation, tachycardia, systemic vasoconstriction and adrenaline release from the adrenal medulla (Kumar, 2009).

There is now a general consensus that a series of key processes contribute to the CB hypoxic chemotransduction cascade. These include attenuation of outward K^+^ current, type I cell depolarization, Ca^2+^ influx through L-type Ca^2+^ channels, neurosecretion and chemoafferent excitation (Kumar and Prabhakar, 2012; Lopez-Barneo et al., 2016). Single type I cells are exquisitely sensitive to O_2_ and rapid (within ms) activation of the hypoxic chemotransduction cascade initiates at PO_2_s of between 20–40 mmHg (Biscoe and Duchen, 1990; Buckler and Vaughan-Jones, 1994; Buckler and Turner, 2013); a level considerably greater than that at which cell metabolism is affected in O_2_-insensitive cells.

What remains highly controversial is the molecular identity of the specific O_2_ sensor within the type I cell. We would argue that the physiological O_2_ sensor should exhibit certain key features: (1) expression in the type I cell permitting intrinsic O_2_ sensitivity; (2) the ability to bind O_2_; (3) its binding of O_2_ occurs over the physiological range at which the type I cell is stimulated; (4) it is required for the CB to be stimulated by hypoxia; and (5) it is able to activate the CB transduction cascade within milliseconds. Many proposed sensors fit one or two of these criteria but few have been shown to adequately comply with all five.

In mammalian cells, O_2_ is the terminal electron acceptor in the mitochondrial respiratory chain. Continuous binding and reduction of O_2_ in the CuB/haem a_3_ (cytochrome a_3_) binuclear center of complex IV drives mitochondrial electron transport and promotes activation of the mitochondrial ATP synthase. The long-established, mitochondrial hypothesis for chemoreception proposes that CB excitation induced by hypoxia is initiated by a reduction in O_2_ dependent mitochondrial energy respiration. This review will briefly critique the current evidence as to whether the mitochondria can be considered the functionally relevant O_2_ sensors within the CB.

## Mitochondrial Inhibitors Mimic All Aspects of the Carotid Body Hypoxic Chemotransduction Cascade

All mitochondrial poisons induce chemoafferent excitation (Krylov and Anichkov, 1968; Mulligan et al., 1981; Obeso et al., 1989; Donnelly et al., 2014; Holmes et al., 2016, 2017) leading to rapid increases in ventilation (Owen and Gesell, 1931), heart rate and arterial blood glucose (Alvarez-Buylla and de Alvarez-Buylla, 1988). Chemoafferent responses are rapid, dose dependent and reversible (Donnelly et al., 2014; Holmes et al., 2016) and the magnitude of the rise in chemoafferent frequency caused by saturating concentrations is consistent with those evoked by severe hypoxia or anoxia (Krylov and Anichkov, 1968; Mulligan et al., 1981; Obeso et al., 1989). Furthermore, mitochondrial inhibitors and uncouplers augment neurotransmitter secretion, confirming an action through the type I cell rather than the afferent nerve endings (Obeso et al., 1989; Rocher et al., 1991). Despite the strong consistency between all of the different types of mitochondrial poisons (both in the older and more recent studies), it should be noted that some of the pharmacological agents used may not have acted selectively on the mitochondria and so the conclusions should be viewed with a certain degree of caution.

Mitochondrial poisons cause fast (within ms) type I cell depolarization and increases in [Ca^2+^]_i_. The size and timing of the [Ca^2+^]_i_ rise observed using many different mitochondrial inhibitors or uncouplers closely resembles that seen in hypoxia (Biscoe et al., 1989; Biscoe and Duchen, 1990; Wyatt and Buckler, 2004; Buckler, 2011). As with hypoxia (Buckler and Vaughan-Jones, 1994; Urena et al., 1994), the increases in [Ca^2+^]_i_ are dependent on cellular depolarization and extracellular Ca^2+^ influx through voltage gated Ca^2+^ channels (Wyatt and Buckler, 2004). TASK1/3, TREK-1, BK_Ca_, K_v_4.1, and K_v_4.3, have all been shown to be expressed in the CB and inhibited by hypoxia (Buckler et al., 2000; Sanchez et al., 2002; Williams et al., 2004; Yamamoto and Taniguchi, 2006; Kim et al., 2009; Kreneisz et al., 2009; Turner and Buckler, 2013; Wang et al., 2017b). Of these, TASK1/3 and TASK-like K^+^ currents are diminished by a multitude of mitochondrial inhibitors leading to membrane depolarization (Barbe et al., 2002; Wyatt and Buckler, 2004; Buckler, 2011; Turner and Buckler, 2013; Kim D. et al., 2015).

Recent evidence has revealed the presence of TRP and other non-selective Ca^2+^-activated cation currents in type I cells that are activated by hypoxia (Kumar et al., 2006; Kang et al., 2014; Kim I. et al., 2015; Wang et al., 2017a). Although intriguing, the full functional relevance of these currents in type I cell O_2_-sensing remains to be further characterized, and in particular whether these currents can be upregulated to preserve O_2_ sensing in the absence of TASK channels (Turner and Buckler, 2013). Current evidence suggests that mitochondrial inhibitors are also capable of increasing these inward depolarizing currents (Kim I. et al., 2015; Wang et al., 2017a).

## Mitochondria are Necessary for the Carotid Body to Respond to Hypoxia

The presence of functional mitochondria does appear necessary for the CB to respond fully to hypoxia. For instance, cyanide, rotenone and FCCP all attenuate TASK channel currents in such a way that prevents any further reduction by hypoxia (Wyatt and Buckler, 2004). In intact CB preparations oligomycin, cyanide and azide all reduce or abolish subsequent chemoafferent responses to hypoxia (Mulligan et al., 1981; Donnelly et al., 2014). Some of the attenuation observed in these experiments may have been due to impairment of oxidative phosphorylation in the chemoafferent fibers, limiting their excitability. Any such impairment is not apparent in response to physiological levels of hypoxia, since the PO_2_ that activates the type I cells occurs at a much higher level than those which would decrease mitochondrial function in the chemoafferent fibers. As such, chemoafferent responses to sustained hypoxia are better maintained than those in response to sustained high doses of mitochondrial inhibitors (Mulligan et al., 1981).

In a recent study the importance of mitochondrial complex I was tested by developing mice deficient in *Ndufs2* (a gene coding for NADH dehydrogenase [ubiquinone] iron-sulfur protein 2- a component of complex I that participates in ubiquinone binding) in tyrosine hydroxylase positive cells (Fernandez-Aguera et al., 2015). Type I cells isolated from these mice were insensitive to hypoxia; they lacked any hypoxia-induced K^+^ current attenuation, [Ca^2+^]_i_ elevation or neurotransmitter release. Furthermore, these mice failed to increase respiratory frequency when breathing 10% O_2_. This work supported a previous study in which type I cell hypoxic chemosensitivity was abolished in the presence of rotenone (Ortega-Saenz et al., 2003). The authors propose a mechanism whereby exposure to hypoxia promotes reverse electron transport and ROS/NADH generation via complex I which is driven by a high rate of succinate oxidation at complex II. Accordingly, they have recently shown that genetic and pharmacological deactivation of complex II completely blocks type I cell hypoxic sensitivity (Gao et al., 2017).

This intriguing and elegant hypothesis does, however, show some discrepancies with evidence from earlier reports. For instance, similar experiments performed on CBs with heterozygous *Sdhd* knock out displayed an augmented, rather than depressed basal activity and had a completely preserved hypoxic response (Piruat et al., 2004). Furthermore, when rat type I cells were exposed to tetramethyl-*p*-phenylenediamine (TMPD) and ascorbate in the presence of rotenone, there was still a robust elevation in Ca^2+^ upon hypoxic stimulation (Wyatt and Buckler, 2004). This would suggest that feeding electrons into cytochrome c is sufficient to sustain type I cell hypoxic sensitivity even when complex I activity (and ROS generation) is inhibited. Complex IV activity rather than complex I and II may therefore be necessary for hypoxic chemotransduction. The same report also showed that application of H_2_O_2_ was unable to excite the type I cell directly. This observation is consistent with the lack of effect of multiple anti-oxidants used in other CB preparations and animal species (Sanz-Alfayate et al., 2001; Agapito et al., 2009; Gomez-Nino et al., 2009). Interestingly, using novel *ex vivo* CB culture techniques combined with FRET based ROS sensors, Bernardini et al. (2015) deduced that type I cell ROS actually decreases in hypoxia due to reduction in NADPH oxidase activity (an alternative ROS source). Clearly there is a need for reconciliation between these findings.

## The Carotid Body Mitochondria are Unique and Have a Low Threshold for O_2_

The evidence that mitochondria are required for CB O_2_ chemotransduction and that mitochondrial inhibitors can cause chemoexcitation, is not enough to define them as the O_2_-sensors in the CB. Clearly, mitochondria are able to bind O_2_. However, the K_m_ of the cytochrome a_3_ for O_2_ is reported to be <1 mmHg in isolated mitochondria and between 1–5 mmHg in dissociated cells and tissue preparations, with little variation existing between different cell types (Wilson et al., 1988; Tamura et al., 1989). This is far lower than the PO_2_ at which the CB type I cells begin to be activated and, for this reason, is a common argument against the mitochondrial hypothesis.

However, there is now a substantial body of evidence indicating that the CB type I cell mitochondria are unique. Experiments performed by Mills and Jobsis (1970, 1972), were the first to identify an unusually low affinity cytochrome a_3_ within the CB. Using absorbance spectra, they estimated that 43–67.5% of total cytochrome a_3_ within the intact CB preparation had a remarkably low O_2_ affinity. This fraction was reported to be almost 100% reduced at PO_2_s between 7–9 mmHg and 50% reduced at a PO_2_ as high as 90 mmHg. In contrast, the remaining fraction was only 50% reduced at a PO_2_ of approximately 0.8 mmHg, comparable to cytochrome a_3_ found in other tissues (Gnaiger, 2001). Thus, the CB appeared to express both low and high affinity subtypes of cytochrome a_3_. At that time, the specific cellular location(s) of each was unclear. Later experiments utilized the photolabile binding of CO, to deduce that saturation of cytochrome a_3_ with CO prevented any additional chemoafferent excitation during hypoxia, implying that not only was the cytochrome a_3_ in the CB unusual, it was also required for O_2_-sensing (Wilson et al., 1994; Lahiri et al., 1999). It should be pointed out that the concentrations of CO used in these studies could have directly modified the activity of the BK_Ca_ channel (Williams et al., 2004, 2008) and the generation of H_2_S (Yuan et al., 2015) and as such some of the observations could be related to mechanisms independent of the mitochondria.

In dissociated rabbit type I cell clusters, mitochondrial electron transport begins to be inhibited at a high PO_2_ value of approximately 40 mmHg (Duchen and Biscoe, 1992a). PO_2_-NADH response curves demonstrate a significant ‘right shift’ in type I cells compared to sensory neurons, indicative of a heightened and distinctive O_2_ sensitivity. In addition, mitochondrial depolarization occurs at higher PO_2_s compared to O_2_-insensitive cells (Duchen and Biscoe, 1992b). More recent work has verified the “right shifts” in both PO_2_-NADH and PO_2_-rh-123 response curves in rat type I cells, confirming that the unusually low mitochondrial O_2_ affinity is conserved in multiple species (Turner and Buckler, 2013). By isolating complex IV activity with a cocktail of mitochondrial inhibitors plus TMPD and ascorbate, the authors were able to reveal that complex IV activity is a component of the mitochondria with the exceptionally low O_2_ affinity. Importantly, type I cell hypoxic response curves for electron transport inhibition, mitochondrial depolarization and complex IV run-down display considerable overlap with the rise in Ca^2+^, indicating that these processes are intimately linked. Therefore, it does appear that type I cell mitochondria have a highly specialized *low affinity* for O_2_ due to an altered function/subtype of cytochrome a_3_ in complex IV that predisposes CB energy metabolism to being impaired at high O_2_ tensions. It is likely that the high affinity cytochrome a_3_ in the CB described by Mills & Jöbsis is located in the non-O_2_ sensing tissue such as the, nerve endings, blood vessels and type II cells.

Understanding the mechanism linking a fall in mitochondrial O_2_ consumption with K^+^ channel inhibition (or cation channel activation) is contentious. As previously mentioned, there could be a role for elevated mitochondrial ROS generation but this is still to be validated (Fernandez-Aguera et al., 2015). Another possibility is an alteration in cytosolic nucleotides. Switching from a cell attached to inside-out patch configuration diminishes background K^+^ channel activity, suggesting that a basal level of an intracellular factor(s) activates TASK channels in normoxia (Varas et al., 2007). Addition of 5 mM MgATP in the inside-out configuration can restore about 50% of this background K^+^ channel activity. Both mitochondrial inhibition and hypoxia also significantly elevate free Mg^2+^, consistent with a decrease in MgATP. Thus, the fall in MgATP during hypoxia is likely to attenuate a significant proportion of TASK-current leading to depolarization. However, the remaining modulators that account for the other 50% of TASK current are still to be identified.

**FIGURE 1:**
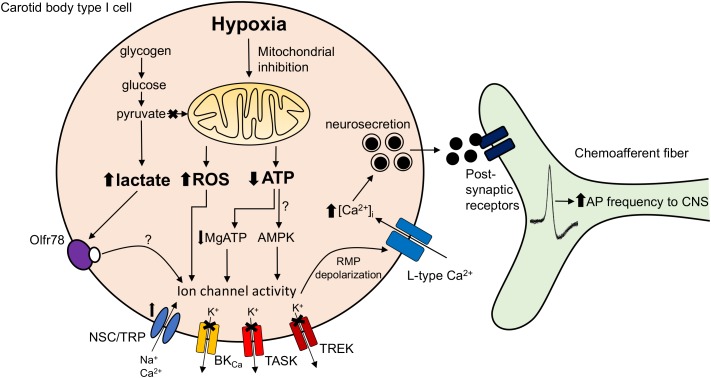
Carotid body mitochondrial signaling mechanisms activated during hypoxia. Hypoxia-induced mitochondrial inhibition is proposed to increase lactate generation (Chang et al., 2015), augment mitochondrial complex I reactive oxygen species (ROS) production (Fernandez-Aguera et al., 2015) or reduce mitochondrial ATP synthesis (Buckler and Turner, 2013). These changes are proposed to directly or indirectly (e.g., via Olf78 receptor activation, reduced MgATP concentration or stimulation of AMP-activated protein kinase; AMPK) modify ion channel function leading to resting membrane potential (RMP) depolarization. This causes opening of L-type Ca^2+^ channels, neurosecretion and an increase in discharge frequency of the adjacent chemoafferent fibers. TRP, transient receptor potential channel; NSC, non-selective cation channel; BK_Ca_, large conductance Ca^2+^-activated K^+^ channel; TASK, TWIK-related acid-sensitive K^+^ channel; TREK, TWIK-related K^+^ channel; AP, action potential.

Another proposed mediator of TASK channel activity that is sensitive to changes in cytosolic nucleotide concentrations is AMPK (Wyatt et al., 2007). However, initial favorable studies based on pharmacological evidence have since been challenged by the finding that the AMPK-α_1_α_2_ deficient CB retains complete O_2_-sensitivity (Mahmoud et al., 2016). Other groups have also shown that pharmacological targeting of AMPK does not impact on the hypoxia-induced K^+^ channel inhibition (Kim et al., 2014). Discrepancies may arise from the non-selectivity of the drugs used to evaluate AMPK function and potential redundancy mechanisms known to develop in genetic animal models (Nowak et al., 1997). A final hypothesis is that a build-up in lactate upon mitochondrial inhibition in hypoxia activates the olfactory receptor Olfr78 (Chang et al., 2015). However, the concentration of lactate necessary to elevate Ca^2+^ in an intact CB preparation appears to be quite high (30 mM) and whether local levels reach this threshold during hypoxia is uncertain. We await a mechanism demonstrating how activation of Olfr78 (a G-protein-coupled receptor) modulates TASK or cation channel activity. A summary of the proposed O_2_-sensitive mitochondrial signaling pathways in the CB is presented in **Figure 1**.

## What Determines the Low O_2_ Affinity of the Carotid Body Mitochondria?

**FIGURE 2:**
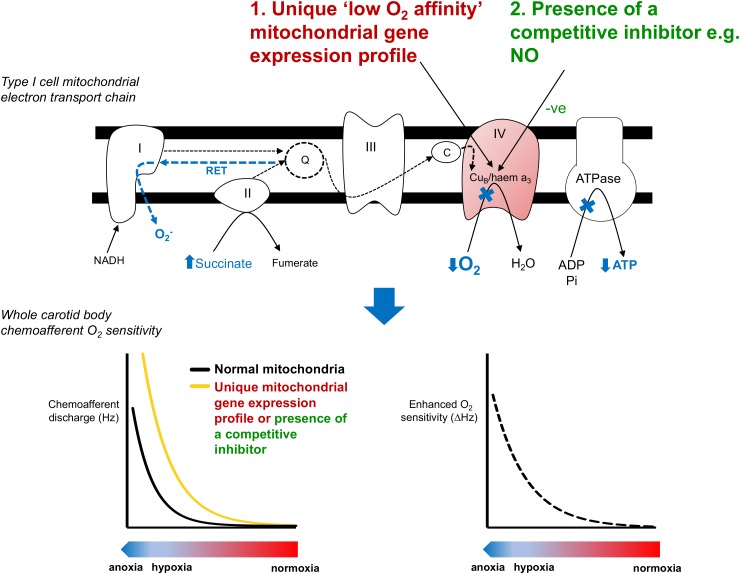
Potential mechanisms underlying the unique low O_2_ affinity mitochondria in the carotid body. Inhibition of mitochondrial function in the carotid body type I cell occurs at PO_2_ values well above those that inhibit metabolism in other O_2_ insensitive cells. This activates a number of proposed signaling pathways (shown in blue). The unique low O_2_ affinity of the carotid body mitochondria could be due to (1) a unique mitochondrial gene expression profile or (2) the presence of a competitive inhibitor such as NO (Holmes et al., 2016). The impact of lower mitochondrial O_2_ affinity is to cause a right-shift in the PO_2_-chemoafferent response curve, thereby enhancing the functional O_2_ sensitivity and allowing the carotid body to respond over a more physiological range of arterial PO_2_s. RET, reverse electron transport.

We propose that there are 2 potential means to account for the extraordinary O_2_ sensitivity of type I cell mitochondria. First, there could be a high level of production of a cytosolic factor that is able to freely diffuse into the mitochondria and then compete with O_2_ binding in complex IV (**Figure 2**). We predict that this competition would render mitochondrial electron transport more susceptible to subsequent falls in O_2_. We recently tested this by applying exogenous nitrite to CBs and subsequently measuring hypoxic sensitivity (Holmes et al., 2016). Nitrite is reduced within the mitochondria to generate local NO, a recognized competitive inhibitor of complex IV (Brown and Cooper, 1994; Cleeter et al., 1994; Castello et al., 2006, 2008; Basu et al., 2008). Moderate basal inhibition of the CB mitochondria by nitrite exaggerated the subsequent chemoafferent excitation during hypoxia signifying an increase in CB O_2_ sensitivity. Therefore, we validated the idea that CB hypoxic sensitivity could be adjusted by a factor capable of competing with O_2_ in the mitochondria and suggested a physiological role for endogenous NO in establishing type I cell mitochondrial O_2_-sensitivity. Measurable amounts of NO have been detected in mitochondrial of type I cells (Yamamoto et al., 2006). A possible source is nitric oxide synthase 3 (NOS-3) given its location within the type I cell (Yamamoto et al., 2006). Interestingly, mice with reduced NOS-3 have a dampened hypoxic ventilatory response and a depressed CB function (Kline et al., 2000). One explanation for this is an adaptation to chronic hypoxia brought about by reduced CB blood flow. However, this is unlikely as there is no significant type I cell hyperplasia/hypertrophy (McGregor et al., 1984; Tatsumi et al., 1991). Instead, the blunted CB activity could be due to the lack of NO acting on the CB mitochondria. Consideration of the precise compartmentalization of NO should also be taken into account. Whilst NO in the mitochondria induces chemostimulation, its action in other regions is likely to have opposing effects via modulation of soluble guanylate cyclase and L-type Ca^2+^/BK_Ca_ channels (Summers et al., 1999; Iturriaga et al., 2000; Silva and Lewis, 2002; Valdes et al., 2003). In addition, other diffusible cytosolic factors have been implicated in CB O_2_ sensing including H_2_S and CO (Peng et al., 2010; Yuan et al., 2015). Both of these gasses are capable of inhibiting type I cell mitochondria (Wilson et al., 1994; Lahiri et al., 1999; Buckler, 2011). Future experiments are required to evaluate if these substances act by setting type I cell mitochondrial O_2_ sensitivity.

A second explanation for the low O_2_-affinity of the type I cell mitochondria is that it has a unique gene expression profile (**Figure 2**). Exploring gene expression in the CB is challenging due to its relatively small size and heterogeneity. However, advances in molecular biology techniques now make it possible to perform whole genome analysis using just micrograms of tissue or even single cells. RNA-sequencing analysis has now revealed a number of mitochondrial related genes that have a particularly high expression in the type I cell (Zhou et al., 2016). Of these *Ndufa4l2* and *Cox4i2* have been shown to be more highly expressed in the CB compared with tissue from the superior cervical ganglion (Gao et al., 2017). Whether these two genes contribute to the low mitochondrial O_2_ affinity remains to be determined but these findings do support the idea that CB mitochondria have a unique genetic signature encoding their mitochondrial complexes. We would expect many more genetic studies to probe this further until the type I cell mitochondria can be accurately modeled to pinpoint the structural conformation underlying its low O_2_ affinity. An interesting comparator may be the mitochondria isolated from guinea pig CB which does not appear to have any inherent O_2_ sensitivity (Gonzalez-Obeso et al., 2017).

## Conclusion

On current evidence, it is very hard to disprove the mitochondrial hypothesis of CB O_2_ sensing. The mitochondria seem to fulfill all five criteria that we have proposed for adequate O_2_-sensors. What is less clear is a mechanistic understanding of how the low O_2_ sensitivity of the CB mitochondria is achieved and if mitochondria are involved in establishing pathological changes in CB function.

## Author Contributions

AH, CR, AC, and PK all contributed to the writing and editing of the manuscript.

## Conflict of Interest Statement

The authors declare that the research was conducted in the absence of any commercial or financial relationships that could be construed as a potential conflict of interest.
